# Predicting the evolution and control of the COVID-19 pandemic in Portugal

**DOI:** 10.12688/f1000research.23401.2

**Published:** 2020-09-09

**Authors:** Ricardo J. Pais, Nuno Taveira

**Affiliations:** 1 Centro de investigação Interdisciplinar Egas Moniz (CiiEM), Instituto Universitário Egas Moniz, Caparica, 2829-511, Portugal; 2 Research Institute for Medicines (iMed.ULisboa), Faculty of Pharmacy, University of Lisbon, Lisbon, 1649-003, Portugal

**Keywords:** COVID-19, Pandemic Control, Predictive modeling, Simulation, Social Isolation, Mathematical model

## Abstract

Coronavirus disease 2019 (COVID-19) is a worldwide pandemic that has been affecting Portugal since 2 March 2020. The Portuguese government has been making efforts to contradict the exponential growth through lockdown, social distancing and the usage of masks. However, these measures have been implemented without controlling the compliance degree and how much is necessary to achieve an effective control. To address this issue, we developed a mathematical model to estimate the strength of Government-Imposed Measures (GIM) and predict the impact of the degree of compliance on the number of infected cases and peak of infection. We estimate the peak to be around 650 thousand infected cases with 53 thousand requiring hospital care by the beginning of May if no measures were taken. The model shows that the population compliance of the GIM was gradual between   30% to 75%, contributing to a significant reduction on the infection peak and mortality. Importantly, our simulations show that the infection burden could have been further reduced if the population followed the GIM immediately after their release on 18 March.

## Introduction

Coronavirus disease 2019 (COVID-19) is already considered a world pandemic which is starting to have dramatic effects in Europe, where, as of 27 of March, 265,421 cases have been reported ^1, 2^. COVID-19 infection in Portugal has been growing exponentially with an average rate of 34±13% new cases per day from 2 March and is far from reaching the peak by the end of March. As of March 27, 4268 infection cases and 76 deaths have been reported ^2^. The highest infection burden is found in Porto (317 cases, 7.4%) and in Lisbon (284 cases, 6.7%) but the disease is present throughout the entire country. As in other countries, infection occurs mostly in individuals’ with ≥40 years of age (71.9% males; 69.3% females). Death occurs mostly in males (64.5%) all with ≥50 years of age. 

Predictive models estimate that the peak of SARS-CoV-2 infection globally would be between mid-April and May, with an estimated total of 48 million people infected ^3^. As with most other countries, the Portuguese national health care system cannot deal with the increasing demand of care due to limited ventilators and care units ^3^. Therefore, the Portuguese government together with the National Health Directorate (DGS) declared a state of emergency and adopted interventive populational measures through Government-Imposed Measures (GIM) on 18 March 2020 in an attempt to drop the peak of infections even if at the cost of prolonging the infection time. These measures are based on the lockdown of people at home, social distancing and adopting protective antiseptic policies such as the usage of masks. Lockdown was implemented to assure compliance of the population, expect for people that maintain basic services such as medical and food distribution staff. 

Most forecasting models are based on the number of cases reported and do not take into account the effects of these government-imposed measures and behavioral change. Thus, accessing the compliance degree and predicting how much is necessary for the control of SARS-CoV2 infection would be a useful tool for fighting COVID-19 pandemic. Recently published mathematical modelling studies of COVID-19 transmission have already provided useful insights that can be used to guide public health measures and resource allocation to better control this pandemic ^4– 7 ^. However, most parameters of statistical models have been estimated with high degree of uncertainty, resulting in predictions with wide intervals of confidence ^4, 6^. Compartmental models such as susceptible, infected and resistant (SIR) models are deterministic approaches based on solving nonlinear systems of Ordinary Differential Equations (ODE) that have been successful in describing complex dynamics of virus infection in populations, including COVID-19 in several countries ^7– 11^. Here, we provide a simple SI model that describe the dynamics of transition of COVID-19 in Portugal during the first 21 days, explain the evolution of SARS-CoV-2 infection dynamics up to 19 of August and predicts the degree of compliance of GIM by the Portuguese population.

## Methods

Basic transmission dynamics of COVID-19 was modelled using a simple mathematical model based on a system of two ordinary differential equations (ODE) developed specifically for this purpose ( Equation 1 and Equation 2). The equations reflect the number of people infected ( *I*) and susceptible ( *S*) to infection per unit of time ( *dI*/ *dt* and *dS*/ *dt*). In this model, we accounted for the reported average time of duration of infection ( *τ*) of 14 days ^4, 11^. The model was calibrated by adjusting the rate constant ( *k*) to approximate the total infection value reported by the DGS at 17 March. No further fitting was performed in this model. The compliance of GIM by different fractions of the population was modelled through the variation of parameter *α* in Equation 1 and Equation 2. We considered that these protective measures (GIM) were 97% effective based on recent meta-analysis estimates, accounted through model parameter *β* ^12^. The ODEs were encoded and solved using PLAS software version 1.2.0.120, where a series of simulations were carried scanning various values of the α parameter ^13^. Simulations were carried with the initial two cases reported by the DGS and considering only the population of the grand Lisbon and Porto areas (total of 6.5 × 10 ^6^) since they represent most of the susceptible population (see Figure 2). For simulations, we used the numerical solver based on the Adams/BDF method, implemented in the LSODA routine of PLAS software. Because a serological screening study made by the Portuguese Nacional Institute of Health ( http://www.insa.pt/) found a 6-fold higher infected due to untested asymptomatic exposed to SARS-CoV-2, we have considered this ratio to estimate the reported symptomatic infected by the DGS. Further analysis, computations and plots were conducted using Python 3 in the Jupiter Notebook ipython 7.8.0 programing environment under Anaconda distribution version 4.7.12. Data regarding the daily evolution of number of total infected in Portugal by COVID-19 was collected from the DGS web site ( https://covid19.min-saude.pt/ponto-de-situacao-atual-em-portugal/) from 2 March to 19 August 2020 (see *Source data*, Table S1 and Figure S1) ^14^. The model is available as *Extended data*.

$$\frac{dI}{dt}=k(1-\alpha )SI+\alpha k\beta SI-\frac{1}{\gamma }I\;(\text{Equation}\;1)$$

$$\frac{dS}{dt}=-k(1-\alpha )SI-\alpha k\beta SI\;(\text{Equation}\;2)$$

## Results and discussion

**Figure 1.**
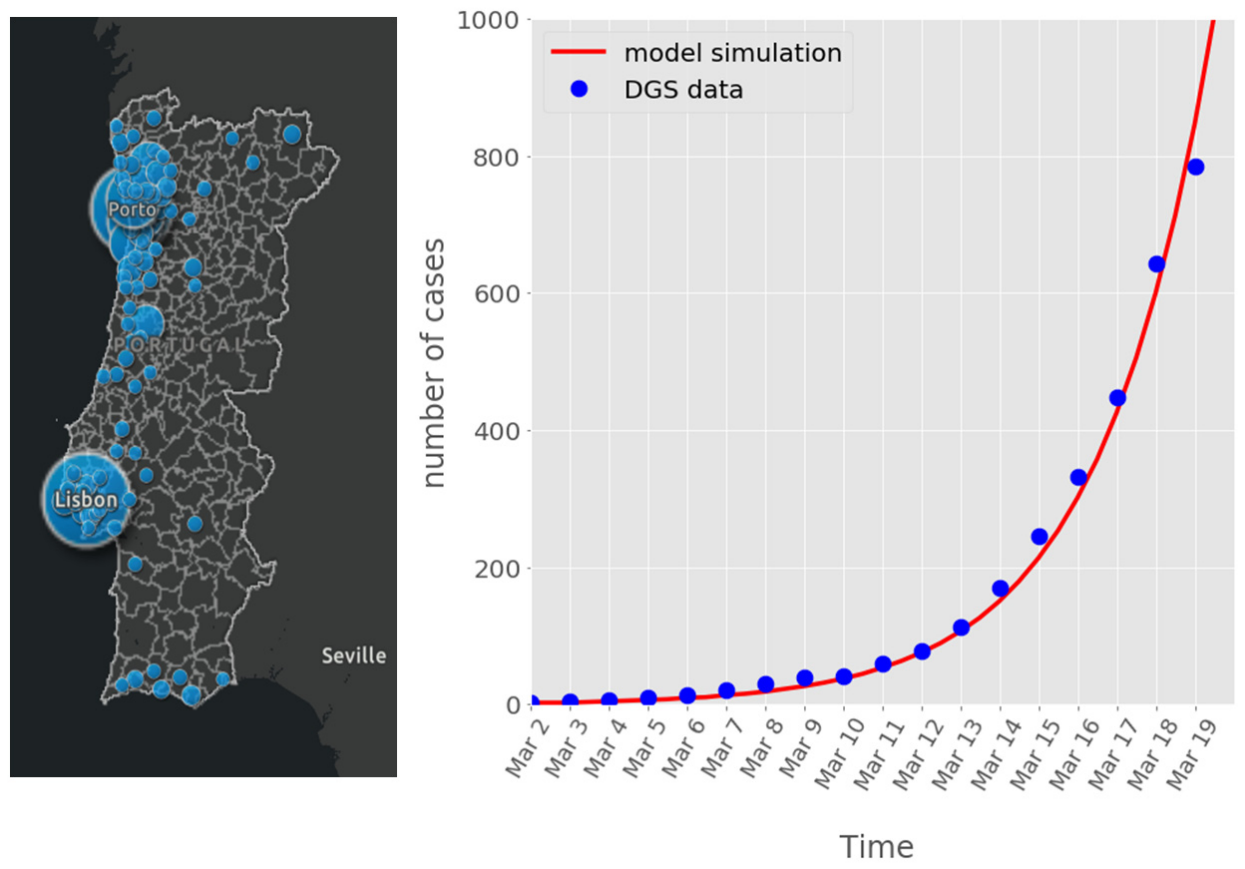
COVID-19 spreading on Portuguese population up to 19 of March. Left, the distribution of confirmed cases on 19 March are depicted in the map. Right, evolution of the cases between 2 and 19 of March. Lines indicate simulation using the mathematical model and blue dots correspond to the confirmed cases reported by DGS.

Simulation of the first 18 days with our model was able to describe the exponential increase of the number of confirmed cases reported by the DGS between 2 and 18 March 2020 ( Figure 1). The predicted peak time for this scenario was 49 days which would be by the 21 of April. This is within the estimated range predicted by statistical modelling of US, Italy and Korea scenarios ^3^. Further, the predicted numbers of cases for the end of March if no measures were taken would be around 42,000. This is also in agreement with the number released by the DGS to the social media based on statistical modelling. Thus, the model presented here is consistent with the forecasting made by conventional models, reinforcing the confidence on our model capacity to generate predictions.

**Figure 2.**
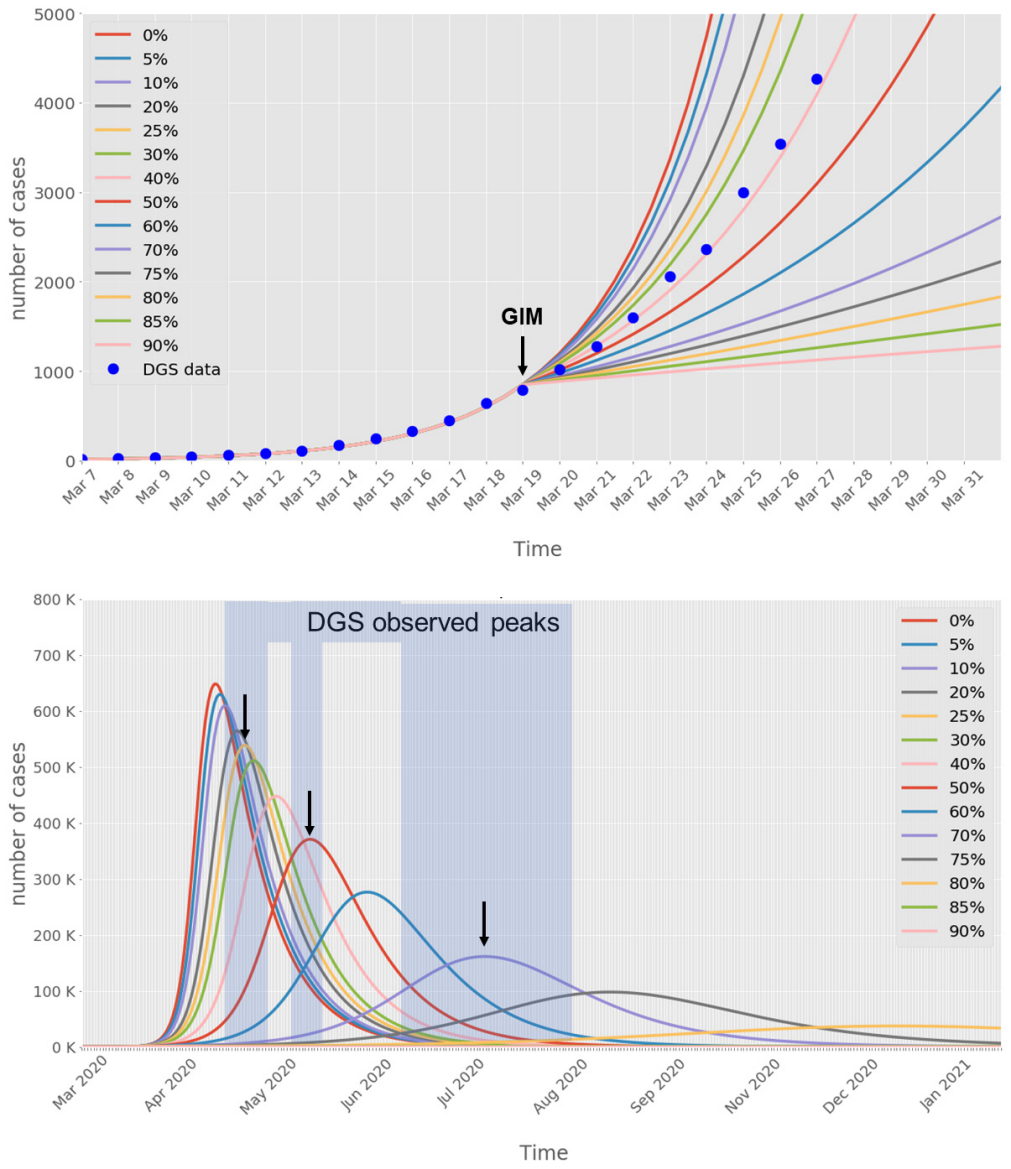
Simulation of the transmission dynamics of SARS-CoV-2 infection on Portuguese population with different percentages of compliance of Government-Imposed Measures (GIM). Above, predicted total infected population in the month of March. The starting of the measures is depicted by GMM and the arrow indicates the time of change. Below, Predicted peak of infection. Observed peaks of SARS-CoV-2 infection by the DGS are indicated by arrows and their intervals in blue. These peaks were collected based on reported new cases and hospitalizations due to SARS-CoV-2 infection up to 19 August 2020 (available in figure S1 as extended data ^14^).

 Importantly, our results show that the GIM had an immediate impact on diminishing the exponential increase of the number of infected cases and this depends on the percentage of the population that is in compliance with such measures ( Figure 2). This is evident by the increasing deviation of the reported number of cases relative to the unperturbed simulation (0%) with time. The evolution of the number of cases reported by DGS between 18 and 25 March fit between the simulation curves corresponding to 30% and 40% of model perturbation on parameter *α* This suggests that the estimated percentage of the Portuguese population that have been start following the GIM was between 30% to 40%. From simulations, we identify other intervals (e.g. 50–60% and 70–75%) that are compatible with the reported data form DGS between April to August 2020, regarding observed peaks of infection and hospitalizations ( Figure 2, Table 1). For 19 august, the computed total infections and deaths for the 70–75% interval is 30,664 - 91,426 and 1,004 - 2,995, respectively. This is within the range of the reported values by the DGS for this day (54,701 and 1,786, respectively) ^14^, making our model consistent with the reported data by the DGS. Together, these results indicate that GIM compliance degree shifted from 30–40% to 70–75% suggesting a gradual compliance degree of the Portuguese population.

**Table 1.** Predicted ranges (upper and lower values) for several SARS-CoV-2 infection indicators under three GIM compliance scenarios (percentages).

Indicators	30–40%	70–75%	90%
Total Infected	885,725 – 928,703	456, 446 – 572,624	847
Total death	14,674 – 16,754	5,288 – 9,056	23
Infected (on peak)	447,876 – 511,341	97,877 – 161,413	702
Hospitalized (on peak)	37,148 – 42,412	8,118 - 13,388	58
Expected peak occurrence	26 Apr – 4 May 2020	10 Jul – 20 Aug 2020	No peak.

Based on the fraction of hospitalized and mortality reported by the DGS on 27 March 2020, together with our model predictions, we computed several infection indicators for these intervals ( Table 1).

Our model analysis indicates that current government-mandated measures together with compliance changes shifted at least two times the expected peak of infections, causing a substantial reduction in the infection numbers ( Figure 2, Table 1). Based on our model, the predicted peak in the number of cases without any interventive measures would be around 650 thousand, whereas current degree of compliance (70–75%) have resulted in a decrease around one half of expected cases, hospitalizations and deaths ( Table 1) Because we used a 6-fold ratio for asymptomatic infected individuals that have not went through testing pipeline, the total infected up to the end of 2020 is estimated to be around 2.7 –3.4 million people, assuming a 70–75% tendency of GIM compliance. This corresponds to about 1/3 of Portuguese population, suggesting that GIM should continue in 2021 to prevent a secondary outbreak. Our simulations also indicate that the SARS-CoV-2 infection could be further reduced if the population had a degree of compliance over 90% starting from 17 of March ( Table 1, Figure 2). This scenario would result in much less total mortality and hospitalization requirements on peak in comparison to the current trend ( Table 1, Figure 2). Meanwhile, percentages >75% comes with the burden of prolonging the time of pandemic control over a year, which can be economically unbearable. Thus, the ideal solutions would be between 70–90% compliance of the GIM. The results obtained during simulations are available as *Extended data*, Table S2 ^14^.

Although our model precisely described the exponential curve and explains the shift in the temporal evolution of DGS data, it has limitations that may compromise the exact values of predictions. The fact that we only assume two compartments (susceptible and infected) considering the main populated cities (Lisbon and Porto) as one is huge approximation that neglects regional dynamics. Thus, the model is just an approximation that reflects an average trend and may fail to explain regional observations. In this model we also neglected many important parameters of infection transmission such as age groups, types of social interactions, contact dependent probability, and viral load dependent probability ^15^. The inclusion of these parameters would definitely make the model more realistic. However, this data is not available for the Portuguese case and these models require accurate processing of data curation for suitable validation. We have bypassed these limitations by aggregating all of these parameters into one constant, which was fitted to the available data. Overall, the predictions shown here should be taken as semi-quantitative estimates within an upper and lower case-scenario.

## Conclusions

In this work we demonstrate the potential of modelling the dynamics of SARS-CoV-2 infection as a useful support tool for predicting the impact of corrective measures as well as estimating the degree of compliance of the GIM by the population. Government-mandated measures on the Portuguese population effectively prevented COVID-19 from reaching dramatic numbers in Portugal but still could be substantially improved to reduce the infection peak. Our estimates and approach may help in guiding additional measures to control the COVID-19 evolution and future epidemies. 

## Data availability

### Source data

Figshare: Modelling COVID-19 evolution and control in Portugal: Code and data from 2 to 27 of March 2020. https://doi.org/10.6084/m9.figshare.12136446.v1 ^14^.

This project contains the following source data used in the present study:

- Table S1 (CSV). (The number of confirmed cases in Portugal officially reported by the DGS.)

### Extended data

Figshare: Modelling COVID-19 evolution and control in Portugal: Code and data from 2 to 27 of March 2020. https://doi.org/10.6084/m9.figshare.12136446.v1 ^14^.

This project contains the following extended data:

- model_code (TXT). (Code used for the model.)
- Table S2 (CSV). (Results obtained during simulation.)
- Python-code (MD). (Python code used with this model.)

Data are available under the terms of the Creative Commons Attribution 4.0 International license (CC-BY 4.0).

## References

[1] WHO: Coronavirus disease 2019 (COVID-19) Situation Report - 62.2020 Reference Source

[2] WHO: Coronavirus disease 2019 (COVID-19) Situation Report - 67.2020 Reference Source

[3] Christopher JLM: Forecasting COVID-19 impact on hospital bed-days, ICU-days, ventilator-days and deaths by US state in the next 4 months.2020 Reference Source

[4] Kucharski AJ Russell TW Diamond C: Early dynamics of transmission and control of COVID-19: a mathematical modelling study. *Lancet Infect Dis.* 2020;20(5):553–558. 10.1016/S1473-3099(20)30144-4 32171059 PMC7158569

[5] Chen TM Rui J Wang QP: A mathematical model for simulating the phase-based transmissibility of a novel coronavirus. *Infect Dis Poverty.*Infectious Diseases of Poverty;2020;9(1): 24. 10.1186/s40249-020-00640-3 32111262 PMC7047374

[6] Yousaf M Zahir S Riaz M: Statistical analysis of forecasting COVID-19 for upcoming month in Pakistan. *Chaos Solitons Fractals.* 2020;138:109926. 10.1016/j.chaos.2020.109926 32501377 PMC7247520

[7] Shah K Abdeljawad T Mahariq I: Qualitative Analysis of a Mathematical Model in the Time of COVID-19. * Biomed Res Int.* 2020;2020:5098598. 10.1155/2020/5098598 32596319 PMC7273369

[8] Abdo MS Shah K Wahash HA: On a comprehensive model of the novel coronavirus (COVID-19) under Mittag-Leffler derivative. *Chaos Solitons Fractals.* 2020;135:109867. 10.1016/j.chaos.2020.109867 32390692 PMC7205740

[9] Din RU Shah K Ahmad I: Study of transmission dynamics of novel COVID-19 by using mathematical model. *Adv Differ Equ.* 2020;2020(1):323. 10.1186/s13662-020-02783-x 32834812 PMC7327217

[10] Huppert A Katriel G: Mathematical modelling and prediction in infectious disease epidemiology. *Clin Microbiol Infect.*European Society of Clinical Infectious Diseases;2013;19(11):999–1005. 10.1111/1469-0691.12308 24266045

[11] Chu DK Akl EA Duda S: Physical distancing, face masks, and eye protection to prevent person-to-person transmission of SARS-CoV-2 and COVID-19: a systematic review and meta-analysis. *Lancet.* 2020;395(10242):1973–1987. 10.1016/S0140-6736(20)31142-9 32497510 PMC7263814

[12] Li Q Guan X Wu P: Early Transmission Dynamics in Wuhan, China, of Novel Coronavirus-Infected Pneumonia. *N Engl J Med.* 2020;382(13):1199–207. 10.1056/NEJMoa2001316 31995857 PMC7121484

[13] Eberhard OV: Computational Analysis of Biochemical Systems: A Practical Guide for Biochemists and Molecular Biologists. Cambridge University Press;2000 Reference Source

[14] Pais RJ Taveira N: Modelling COVID-19 evolution and control in Portugal: Code and data from 2 to 27 of March 2020. *figshare.*Online resource.2020 10.6084/m9.figshare.12136446.v1

[15] Del Valle SY Hyman JM Hethcote HW: Mixing patterns between age groups in social networks. *Soc Networks.* 2007;29(4):539–54. 10.1016/j.socnet.2007.04.005

