# Supplementary material for: Predicting the evolution and control of the COVID-19 pandemic in Portugal
Source: F1000Res. 2020 Sep 9;9:283. Originally published 2020 Apr 23. [Version 2] doi: 10.12688/f1000research.23401.2 (PMC7503179; doi:10.12688/f1000research.23401.2)
Supplement: Peer review history

# Peer review: Reviewer response for version 2

**DOI:** 10.5256/f1000research.29123.r71065

**Published:** 2020-09-18

**Authors:** Elves Heleno Duarte

**Affiliations:** 1 Department of Genetics, University of Cambridge, Cambridge, UK

Pais & Taveira made an outstanding effort and addressed concerns raised by both reviewers. The current version (v2) is, therefore, a significant improvement over the previous version.

 Notably, the authors now conveyed the idea of estimating the strength of the lockdown in Portugal, during early COVID-19 pandemic. Moreover, the authors improved simulations by taking the estimated number of asymptomatic cases into account. Also, they discussed their predicted number of SARS-CoV-2 infection in the light of newly published data by the Portuguese authorities.

 It would be great if the authors could define (biologically or epidemiologically) the parameters A (alpha) and B (beta) (equations 1 and 2). The authors should include, if possible, a citation to the sentence 'This is also in agreement with the number released by the DGS to the social media based on statistical modelling'. The closing punctuation is missing after 'The evolution of the number of cases reported by DGS between 18 and 25 March fit between the simulation curves corresponding to 30% and 40% of model perturbation on parameter α'.

 Finally, I advise re-writing the sentence 'For 19 august, the computed total infections and deaths for the 70–75% interval is 30,664 - 91,426 and 1,004 - 2,995, respectively.' The current version is quite confusing as readers may interpret '70–75% interval' as the confidence interval. It should be clear that these values represent the percentage of lockdown.

Is the work clearly and accurately presented and does it cite the current literature?

Yes

If applicable, is the statistical analysis and its interpretation appropriate?

Partly

Are all the source data underlying the results available to ensure full reproducibility?

Yes

Is the study design appropriate and is the work technically sound?

Partly

Are the conclusions drawn adequately supported by the results?

Partly

Are sufficient details of methods and analysis provided to allow replication by others?

Yes

Reviewer Expertise:

Genetics, bioinformatics, epidemiology, medical entomology, Wolbachia, and host-microorganism interactions

I confirm that I have read this submission and believe that I have an appropriate level of expertise to confirm that it is of an acceptable scientific standard.

# Peer review: Reviewer response for version 2

**DOI:** 10.5256/f1000research.29123.r71064

**Published:** 2020-09-14

**Authors:** Kamal Shah

**Affiliations:** 1 Department of Mathematics, University of Malakand, Chakdara, Chakdara, Pakistan

The authors have revised their paper correctly. I recommend its indexing.

Is the work clearly and accurately presented and does it cite the current literature?

Yes

If applicable, is the statistical analysis and its interpretation appropriate?

Yes

Are all the source data underlying the results available to ensure full reproducibility?

Yes

Is the study design appropriate and is the work technically sound?

Yes

Are the conclusions drawn adequately supported by the results?

Yes

Are sufficient details of methods and analysis provided to allow replication by others?

Partly

Reviewer Expertise:

NA

I confirm that I have read this submission and believe that I have an appropriate level of expertise to confirm that it is of an acceptable scientific standard.

# Peer review: Reviewer response for version 1

**DOI:** 10.5256/f1000research.25829.r64347

**Published:** 2020-07-09

**Authors:** Kamal Shah

**Affiliations:** 1 Department of Mathematics, University of Malakand, Chakdara, Chakdara, Pakistan

Coronavirus disease 2019 (COVID-19) is a worldwide pandemic that has been affecting Portugal since 2 March 2020. The Portuguese government has been making efforts to contradict the exponential growth through social isolation measures. In this regard, the authors have developed a mathematical model to predict the impact of such measures in the number of infected cases and peak of infection. They have estimated the peak to be around 2 million infected cases by the beginning of May if no additional measures are taken. The model shows that current measures effectively isolated 25-30% of the population, contributing to some reduction in the infection peak. Importantly, their simulations showed that the infection burden can be further reduced with a higher isolation degree, providing information for a second intervention. The considered study is interesting in this regard and has the potential to give some sound information about COVID-19.

 The work is good but there are some issues to be addressed:

1. Provide the existence of the model.
2. What is the feasible region for the considered model?
3. Also, simulate the model for a long time that is months, 40 days, etc.
4. What techniques for numerical simulation have been used?
5. Some relevant and recent work in this regard also must be included. Please see these examples of recent work:
  On a comprehensive model of the novel coronavirus (COVID-19) under Mittag-Lefer Chaos, Solitons and Fractals xxx (xxxx) 109867 ^1^
  Qualitative Analysis of a Mathematical Model in the Time of COVID-19 , BioMed Research International 2020, Article ID 5098598, 11 pages ^2^
  Statistical analysis of forecasting COVID-19 for upcoming month in Pakistan, Chaos, Solitons and Fractals 138 (2020) 109926 ^3^
  Study of Transmission Dynamics of Novel COVID-19 by Using Mathematical Model." (2020). Archive. ^4^

I recommend its publication in this journal strongly.

Is the work clearly and accurately presented and does it cite the current literature?

Yes

If applicable, is the statistical analysis and its interpretation appropriate?

Yes

Are all the source data underlying the results available to ensure full reproducibility?

Yes

Is the study design appropriate and is the work technically sound?

Yes

Are the conclusions drawn adequately supported by the results?

Yes

Are sufficient details of methods and analysis provided to allow replication by others?

Partly

Reviewer Expertise:

Applied mathematics, Numerical solutions. Mathematical modeling and analysis.

I confirm that I have read this submission and believe that I have an appropriate level of expertise to confirm that it is of an acceptable scientific standard, however I have significant reservations, as outlined above.

## References

[1] : On a Comprehensive Model of the Novel Coronavirus (COVID-19) Under Mittag-Leffler Derivative. *Chaos Solitons Fractals*.2020; 10.1016/j.chaos.2020.109867 109867 10.1016/j.chaos.2020.109867 32390692PMC7205740

[2] : Qualitative Analysis of a Mathematical Model in the Time of COVID-19. *Biomed Res Int*.2020;2020: 10.1155/2020/5098598 5098598 10.1155/2020/5098598 32596319PMC7273369

[3] : Statistical analysis of forecasting COVID-19 for upcoming month in Pakistan. *Chaos Solitons Fractals*.2020;138: 10.1016/j.chaos.2020.109926 109926 10.1016/j.chaos.2020.109926 32501377PMC7247520

[4] : Study of transmission dynamics of novel COVID-19 by using mathematical model. *Advances in Difference Equations*.2020;2020(1) : 10.1186/s13662-020-02783-x 10.1186/s13662-020-02783-x PMC732721732834812

# Response

**Published:** 2020-08-25

**Authors:** Ricardo Pais

**Affiliations:** Centro de investigação Interdisciplinar Egas Moniz (CiiEM), Instituto Universitário Egas Moniz, Portugal

We would like to acknowledge the reviewer for the relevant comments and suggestions.  We have considered them all and have revised the manuscript accordingly. 

Comment 1

"According to the authors, the model 'predicts the impact of isolation measures towards the expected peak of infection'. I am not convinced this is accurate. I believed the authors used the model to estimate the strength of the measures (i.e. percentage lockdown) and not to estimate the impact of the measures on the spread of SARS-Cov-2"

Reply 1

We agree with the suggestion and have changed the manuscript to convey the idea of estimating the strength of measures, which include the percentage of lockdown, social isolation and usage of masks (see revised version). 

Comment 2

"the consistently wrote 'transmission dynamics of COVID-19' and not the correct form 'transmission dynamics of SARS-CoV-2"

Reply 2

We agree with the suggestion and have changed the manuscript replacing COVID-19 with SARS-CoV-2 (see revised version).

Comment 3

"The authors predicted 2 million cases, which I find surprisingly high. Using the optimal isolation percentage (70-75%), the model still predicted over 150,000 cases which are approximately 5-fold higher than the current number of cases in Portugal (~35,000)"

Reply 3

Indeed, our predictions are higher than the reported values. This is because only a small fraction of SARS-COV-2- infected individuals are tested for the virus has only a few patients show symptoms. However, our results are in agreement with  the recent serologic study conducted by the National Institute of Health (INSA) from Portugal that found that a total of 300.000 people were exposed to the virus, 6-fold higher than the number of reported cases.  By the time we conducted the model calibration and analysis, these results were not known, resulting in the deviation between predictions and reported values. Thus, we have corrected the model accounting for the asymptomatic fraction not tested and other recent data. This resulted in novel results which were compared with new data up to August and presented in the revised manuscript.  

Comment 4

"I would like to see the model's prediction using even higher percentages of isolation. This should be also discussed."

Reply 4

We have simulated the model with higher percentages  and discuss the results (see revised manuscript).

# Response

**Published:** 2020-08-25

**Authors:** Ricardo Pais

**Affiliations:** Centro de investigação Interdisciplinar Egas Moniz (CiiEM), Instituto Universitário Egas Moniz, Portugal

We would like to acknowledge the reviewer for the relevant comments and suggestions.  We have considered them all and have revised the manuscript accordingly. 

Comment 1

"Provide the existence of the model"

Reply 1

According to F1000 Research  rules for supplementary data, we included the model code, data and the analysis code in python as extended on figshare (see revised version references).   

Comment 2

"What is the feasible region for the considered model?"

Reply 2

The recommended feasible region for this model is 10 ^8^ > (S+I) > 10 ^4^ where initial S + I =  city population.     

Comment 3

"Also, simulate the model for a long time that is months, 40 days, etc."

Reply 3

We have performed simulations for 500 days for predictions and 17 days for model calibrations. By the time we submitted the paper no more than 27 days of data was available. Indeed, we only used a 17 days simulation for model calibration to estimate a rate constant in the absence of control. Unfortunately, it is not possible to use more data for model calibration since an uncontrolled lockdown was implemented in Portugal immediately after these 17 days of infection. Using more data would actually result in wrong estimates since many people follow the DGS recommendations but others do not.  This is why we have simulated a total of 500 days with multiple % of lockdown scenarios towards estimating how much % of lockdown during the evolution of COVID pandemic.  However, we now include relevant reported DGS data (up to August) for contrasting with model simulations and predictions (see revised version).     

Comment 4

"What techniques for numerical simulation have been used?"

Reply 4

The numerical solver was based on the Adams/BDF method, implemented in the LSODA routine of PLAS software. This is a general-purpose stiff, variable-step and variable-order solver. We add this information in the methods section (see revised version).  

    

Comment 5 

"Some relevant and recent work in this regard also must be included. Please see these examples of recent work"

Reply 5

We have read your suggestions of new modelling work which are quite relevant and innovative for citing as examples of similar modelling approaches applied in the forecasting of SARS-CoV-2 infection dynamics in Pakistan and Wuhan. Thus, we have included these in the new revised version and other recent work as well.

# Peer review: Reviewer response for version 1

**DOI:** 10.5256/f1000research.25829.r63480

**Published:** 2020-06-09

**Authors:** Elves Heleno Duarte

**Affiliations:** 1 Department of Genetics, University of Cambridge, Cambridge, UK

The work presented by Pais & Taveira is entitled Predicting the evolution and control of the COVID-19 pandemic in Portugal and it aims to describe the spread of SARS-CoV-2 during the first 21 days. The authors used a simple mathematical model assuming 14 days infection period.

 According to the authors, the model 'predicts the impact of isolation measures towards the expected peak of infection'. I am not convinced this is accurate. I believed the authors used the model to estimate the strength of the measures (i.e. percentage lockdown) and not to estimate the impact of the measures on the spread of SARS-Cov-2. This would imply a more elaborated study (e.g. case-control study). Indeed the consistently wrote 'transmission dynamics of COVID-19' and not the correct form 'transmission dynamics of SARS-CoV-2'.

 The authors predicted 2 million cases, which I find surprisingly high. Using the optimal isolation percentage (70-75%), the model still predicted over 150,000 cases which are approximately 5-fold higher than the current number of cases in Portugal (~35,000). I would like to see the model's prediction using even higher percentages of isolation. This should be also discussed.

Is the work clearly and accurately presented and does it cite the current literature?

Yes

If applicable, is the statistical analysis and its interpretation appropriate?

Partly

Are all the source data underlying the results available to ensure full reproducibility?

Yes

Is the study design appropriate and is the work technically sound?

Partly

Are the conclusions drawn adequately supported by the results?

Partly

Are sufficient details of methods and analysis provided to allow replication by others?

Yes

Reviewer Expertise:

Genetics, bioinformatics, epidemiology, medical entomology, Wolbachia, and host-microorganism interactions

I confirm that I have read this submission and believe that I have an appropriate level of expertise to confirm that it is of an acceptable scientific standard, however I have significant reservations, as outlined above.
